# A complex systems perspective on chronic aggression and self-injury: case study of a woman with mild intellectual disability and borderline personality disorder

**DOI:** 10.1186/s12888-024-05836-7

**Published:** 2024-05-21

**Authors:** Daan H. G. Hulsmans, Roy Otten, Evelien A. P. Poelen, Annemarie van Vonderen, Serena Daalmans, Fred Hasselman, Merlijn Olthof, Anna Lichtwarck-Aschoff

**Affiliations:** 1 Behavioural Science Institute, Radboud University, Postbus 9104, Nijmegen, 6500 HE The Netherlands; 2 Pluryn Research & Development, Nijmegen, The Netherlands; 3 Faculty of Behavioural and Social Sciences, University of Groningen, Groningen, The Netherlands

**Keywords:** Aggression, Self-injury, Complex systems, Ecological momentary assessments, Mixed-methods, Mild intellectual disability, Borderline personality disorder, Idiographic, Case study

## Abstract

**Background:**

Challenging behaviors like aggression and self-injury are dangerous for clients and staff in residential care. These behaviors are not well understood and therefore often labeled as “complex”. Yet it remains vague what this supposed complexity entails at the individual level. This case-study used a three-step mixed-methods analytical strategy, inspired by complex systems theory. First, we construed a holistic summary of relevant factors in her daily life. Second, we described her challenging behavioral trajectory by identifying stable phases. Third, instability and extraordinary events in her environment were evaluated as potential change-inducing mechanisms between different phases.

**Case presentation:**

A woman, living at a residential facility, diagnosed with mild intellectual disability and borderline personality disorder, who shows a chronic pattern of aggressive and self-injurious incidents. She used ecological momentary assessments to self-rate challenging behaviors daily for 560 days.

**Conclusions:**

A qualitative summary of caretaker records revealed many internal and environmental factors relevant to her daily life. Her clinician narrowed these down to 11 staff hypothesized risk- and protective factors, such as reliving trauma, experiencing pain, receiving medical care or compliments. Coercive measures increased the chance of challenging behavior the day after and psychological therapy sessions decreased the chance of self-injury the day after. The majority of contemporaneous and lagged associations between these 11 factors and self-reported challenging behaviors were non-significant, indicating that challenging behaviors are not governed by mono-causal if-then relations, speaking to its complex nature. Despite this complexity there were patterns in the temporal ordering of incidents. Aggression and self-injury occurred on respectively 13% and 50% of the 560 days. On this timeline 11 distinct stable phases were identified that alternated between four unique states: high levels of aggression and self-injury, average aggression and self-injury, low aggression and self-injury, and low aggression with high self-injury. Eight out of ten transitions between phases were triggered by extraordinary events in her environment, or preceded by increased fluctuations in her self-ratings, or a combination of these two. Desirable patterns emerged more often and were less easily malleable, indicating that when she experiences bad times, keeping in mind that better times lie ahead is hopeful and realistic.

**Supplementary Information:**

The online version contains supplementary material available at 10.1186/s12888-024-05836-7.

## Background

In residential care for individuals with an intellectual disability, challenging behavior is an often used umbrella term for repeatedly engaging in dangerous or threatening behaviors. These can be outer-directed, like aggression towards people or damaging property, and inner-directed, such as self-injurious behavior [1, 2]. The latter is defined as inflicting deliberate damage on- or destruction of one’s own body tissue with or without suicidal intent, for example by skin cutting, burning, scratching, or ingesting inedible objects [3]. For staff, these behaviors are hard to grasp and sometimes difficult to anticipate. Managing incidents afterwards with freedom restricting measures, such as seclusion or fixation, remains an unwanted and increasingly unaccepted common-practice that is harmful to clients and increases staff stress and turnover [4, 5]. Staff typically describe challenging behaviors as a way the individual communicates unmet “complex needs” [6]. Although group-level research reveals many biological, psychological and social correlates of challenging behavior [2, 7, 8], it remains vague what this often-used adjective “complex” means at the individual level. Research focused on the individual rather than on the group can efficiently advance our understanding of complex phenomena [9]. Therefore, this study provides a unique exploration of patterns of chronic aggressive and self-injurious behaviors in one woman with a mild intellectual disability (MID) and borderline personality disorder (BPD), day-by-day over the course of 560 days.

The overall goal is to obtain an in-depth understanding of when and why challenging behaviors occur, using an analytical strategy inspired by complex systems theory (cf [10]). This complex systems lens differs from the dominant biomedical perspective on psychopathology. That is, from a complex systems perspective psychiatric disorders are not understood as latent entities that cause symptoms through (relatively static) hard-wired biological mechanisms, but as dynamic patterns of behaviors, emotions and cognitions that are formed over time [11, 12]. Complex systems principles have guided individual-specific explorations of dynamics in high-risk young adults [13], people with depression [14–16] and dissociative identity disorder [17, 18]. While these studies all used quantitative timeseries analyses to describe the dynamics, qualitative methods are just as well-suited within a complex systems framework. Central to complex systems theory is a holistic approach to understand the person in their environment [12] and qualitative methods can provide a rich account thereof [19]. The current study therefore offers a holistic and dynamic exploration of a woman with MID and BPD, by employing a mixed-methods strategy with three overarching aims. In the following sections we introduce these three aims step-by-step, with more detailed theoretical background.

### Summarizing daily life

The first step is to qualitatively summarize the complex nature of challenging behavior. From a complex systems perspective, any person is considered a complex system, not just individuals with challenging behavior [12]. It is complex because there is no root cause for the way a person (i.e., system as a whole) feels, thinks, or behaves at certain moments in time. Emotions, thoughts or behaviors emerge from continuous and interdependent exchanges between the system’s internal state and its environment [20]. Complex systems are everchanging, which is why an integrative understanding requires a detailed description of the interplay between the system’s and context elements over a longer period of time. It is therefore necessary to sample personal experiences and contextual influences frequently over time, for example by making use of ecological momentary assessment (EMA). EMA is a method in which someone frequently self-reports on current or very recent behaviors and experiences over time (typically via mobile-phone) [21]. The method is well-established in samples with BPD, but although feasible [22] not often used in MID research. In earlier work involving clients with BPD, momentary self-injury was associated with daily ruminations or heightened negative affect [23]. Other EMA studies found the intensity of anger associated with daily reports of aggression [24]. Such internal experiences (i.e., related to thoughts, emotions, or other behaviors) are the primary focus of most EMA research, but there are few studies that explicitly investigate contextual influences and changes [23]. This is remarkable, because theory indicates that (challenging) behaviors are not only internally driven but are to a large extend elicited by environmental factors [12]. For instance, self-injury, is known to occur more frequently when experiencing interpersonal stress [25]. However, internal factors and the environment differs between persons [26, 27]. Whereas one person’s self-injury may be triggered by an argument with parents, someone else’s work pressure may trigger it. To obtain a holistic summary of the person-environment interplay, we first explore person-specific internal states and environmental factors qualitatively.

### Describing change over time

The second step is to zoom out, quantitatively exploring how these factors are ordered in time on the participant’s 560-day timeline. EMA research typically employs multiple daily self-ratings for 1–3 weeks, but individual accounts of challenging behaviors over longer timeframes are scarce. Some studies used not daily but weekly caretaker-reports of challenging behavioral incidents. These showed that, during a period of 41 weeks, staff of 33 inpatients with MID reported in total 210 aggressive- and 104 self-injurious incidents [28, 29]. Interestingly, 4 of those 33 inpatients were responsible for over half of the 210 aggressive incidents, while a staggering 85% of the 104 self-injurious incidents were from only 2 clients. Few individuals thus account for many incidents, but little is known about the day-to-day temporal patterns of such chronic challenging behaviors over the course of weeks or months.

When a person is tracked over longer periods of time, one can detected phases in which certain behaviors are relatively stable. A single-case study using EMA of a person with a major depressive disorder over almost eight months (239 days) [15] found two distinct phases. The first four months were characterized by consistent low self-reported depressive symptoms. On the 127th day this abruptly changed, marking the start of a four-month period characterized by consistently high depressive symptoms. From a complex systems perspective, these two stable phases (before and after day 127) are called *attractors* [30]. That is, the dynamics of the person (i.e., person-environment system) are attracted towards a specific behavioral pattern that remains relatively stable over time (e.g., a depressive phase in this example). Importantly, stability does not speak to the desirableness of the patterns, but only to the consistency of change over time. For example, consistently never self-harming, consistently being aggressive once-per-week on Tuesdays, or consistently self-harming on weekends are all examples of stable patterns. Following complex systems theory, stable patterns of challenging behaviors can thus be understood as attractors [11, 12]. Our second research question is how challenging behaviors are ordered on the participant’s 560-day timeline? This is done by identifying if there are different attractor states (e.g. time-periods with relatively few vs. many challenging behaviors) and explicate ways in which these time-periods are (dis)similar from one another in terms of internal states (e.g., experienced emotions) and environmental influences (e.g., social interactions).

### Change-mechanisms

In the third and last step we zoom in again by exploring transition-points: moments that ‘kickstart’ abrupt change towards a new attractor (cf. day 127 in [15]). Complex systems theory posits two general mechanisms for the change from one attractor to another that are relevant in the context of this study.

**Fig. 1:**
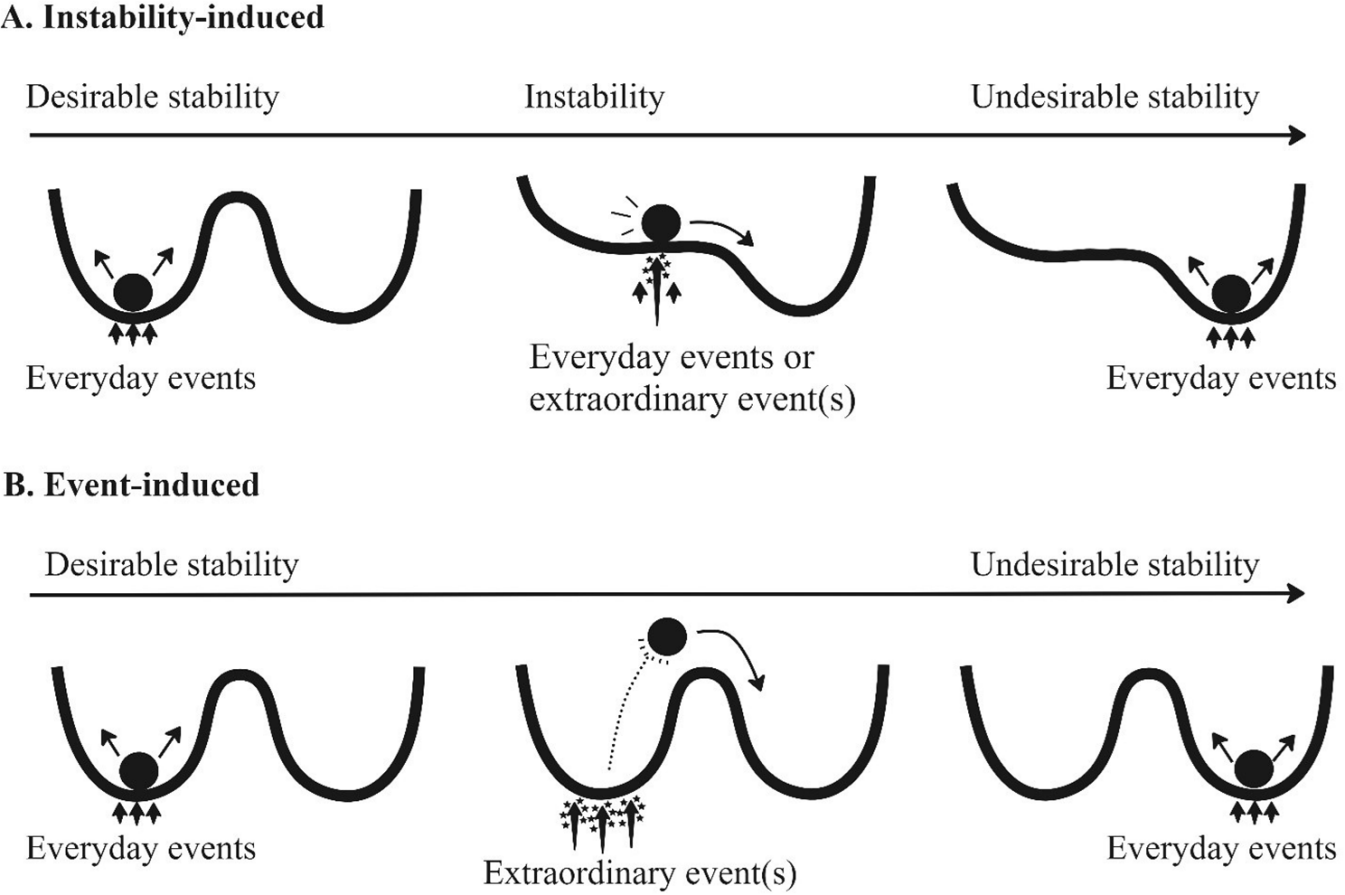
Conceptualization of two potential change-mechanisms according to complex systems theory. Possible attractors are visually conceptualized as a landscape with basins. In this example, the left basin reflects a desirable attractor (few self-injury) and right one an undesirable attractor (frequent self-injury). The ball reflects a person’s state at one point in time while arrows below the ball symbolize interactions between person and environment in daily life. The top panel (**A**) reflects a mechanism in which we can observe instability over time. During instability the attractor loses strength, visualized as the basin becoming more shallow. When this happens, interactions between person and environment, however casual or extraordinary, lead to a transition towards another attractor. The bottom panel’s mechanism (**B**) reflects a mechanism in which the attractor itself does not lose strength. Therefore this will not be marked by instability. Everyday events will not be enough to reach a transition. Instead it takes extraordinarily strong environment-person interaction to ‘force’ this change

First, *instability-induced change* (also called bifurcation-induced change [31]) is the mechanism in which an existing attractor destabilizes, thereby forcing the system to reach a new attractor. In Fig. 1, someone’s current state (e.g., frequently self-injuring) is visualized as a ball, located in a basin which reflects the attractor. The two basins reflect two example attractors: a pattern of few self-injuring behaviors and a pattern of frequent self-injuring behaviors. The basin’s depth metaphorically represents the strength of the attractor state. Stronger attractors are harder to change and therefore everyday events typically do not trigger enduring change. Figure 1A shows instability-induced change, in which an existing attractor destabilizes to the extent that there is no valley left to contain the ball, making the ball roll towards a new valley [11, 12, 32]. Note that during instability, the ball can move more ‘freely’ through the valley (as it is less steep), leading to increasingly variable behavior. Measures of temporal complexity and variability can therefore pick up on instability [33, 34].

Second, *event-induced* (also called noise-induced [31]) change is when an extraordinary event (e.g., unexpectedly being fired from work) ‘pushes’ the ball towards a different attractor, without the existing attractor losing its stability first (Fig. 1B). One would not expect instability as an early warning signal for the transition in this event-induced change, while one would expect the presence of an extraordinary event [12, 31]. This makes it possible to empirically differentiate instability-induced and event-induced changes. The third aim of this study was therefore to evaluate which, if any, of these two change-mechanism(s) potentially underlie transitions between attractors.

## Methods

### Case presentation

The participant is a woman in her 30s, diagnosed with MID and BPD. For over a decade, she has lived in a 24-hour residential care facility specialized for people with MID and severe behavioral problems. Her daily routine typically consists of working in the house (e.g., cooking, cleaning), she likes to take walks, and enjoys playing board games. For several days a week she goes to an activity center where she works creatively (e.g., draw paintings, make music), alone or together with others. This provides important structure in her daily routine. Staff is available 24 − 7 to support her. Even seemingly regular tasks, such as arriving in time for appointments, may be perceived as onerous. Staff are therefore reminded to compliment her regularly, even with seemingly trivial accomplishments. She best thrives when she experiences support that is clear and structured, because that makes her feel calm and secure.

Before she lived in the care facility, during her childhood and teenage years, she experienced traumatic events that undoubtedly contributed to challenges she faces nowadays. She often perceives her life as a struggle, some days more than others. She mostly communicates her struggles calmly to others, but sometimes her tensions become explicit to her environment when she self-injures or is physically aggressive. According to her care professionals, her overall well-being is poorer on days when she shows these behaviors. Her care professionals have several hypotheses about factors contributing to her challenging behaviors. One is that she does not trust herself to be alone. The self-injuring and aggressive incidents are, at least sometimes, perceived as a call for reassuring attention from staff. Another hypothesis is that her challenging behaviors are a maladaptive emotion-regulation strategy. Unpleasant emotions can (sometimes unexpectedly) accumulate very rapidly. Over time, she has learned that she can immediately achieve short-term relief from this overwhelming emotional experience by self-injuring. Alternatively, difficulties regulating negative emotions are also considered a cause of aggressive behaviors. After self-injurious or aggressive incidents, staff need to ensure the participant’s and others’ safety, sometimes by imposing freedom restricting measures such as seclusion or fixation. Such drastic measures are resented by staff and the participant alike. She is highly motivated to change her challenging behavioral patterns, and therefore follows dialectical behavior therapy that aims to increase her emotion regulatory abilities [35].

### Procedure and measures

As part of dialectical behavior therapy, the participant completed daily self-registrations via a mobile phone application. Hence, these EMA data were initially not collected for research purposes. The participant and her clinician formulated the application’s daily EMA questions together. Emotions, behaviors and cognitions with maximum relevance to her treatment goals and daily life were translated into questions that the app prompted automatically on her phone at 7:00 PM. Seven of those questions could be answered on a slider with six answer options that ranged between “not feeling at all” and “an intense feeling”. These questions inquired to what extend she (1) felt happy, (2) felt scared, (3) felt sad, (4) felt angry, (5) had the urge to self-injure, (6) thought of death, and (7) had the urge to be aggressive, on that particular day. She also self-rated with either a “yes” or “no” whether she, on that day, (8) had self-injured and (9) had been physically aggressive. The participant followed dialectical behavior therapy from mid-2019 until mid-2021, which consisted of weekly group sessions with other clients, one-on-one sessions with a therapist and 24-hour telephone consultation. During these individual sessions, therapist and participant discussed recent self-injurious and aggressive incidents registered in the diary. The participant continued to complete her self-ratings on a daily basis, even when therapy was paused due to Covid-19 restrictions. This was not because she was told to – she felt that she benefitted from daily self-reflections in the app. In total, she completed her diaries for a period of 560 days and was rewarded with a gift card for her long-term dedication.

Informed consent was obtained from the participant and her legal guardian to (1) present and analyze the aforementioned daily diary entries and (2) to access the records (i.e., electronic client files) to perform supplementary qualitative analyses about therapeutical context and care professional’s perspective on her functioning. This electronic health system is a routine procedure in which care professionals describe multiple times per day, the provided care, implemented measures and any relevant daily events concerning the participant. The records of the 560-day self-rating period were retrieved and any information that could be traced back (names of persons, cities, organizations, locations) were replaced by codes such ‘Person A’ or ‘City B’. Her clinical team (clinician and closest care professionals) approved aforementioned procedures beforehand. The Ethical Committee Social Sciences of Radboud University and the Ethics committee of the care organization judged that the research was conducted in accordance with the Declaration of Helsinki.

**Fig. 2:**
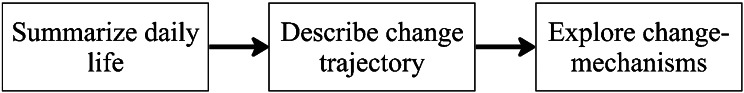
Visualization of our three-step-approach to this case-study

### Design

We employed a mixed-methods triangulation design study with both qualitative and quantitative data [36]. The study had a three-step approach based on complex systems theory (Fig. 2). First, we obtained a comprehensive summary of the participant’s daily life through qualitative analyses of the daily caretaker records. These qualitative findings were then quantified, to then be integrated with quantitative daily self-reports. Secondly, we described the trajectory of her self-reported challenging behaviors by identifying transition-points and characterizing the different attractor states. Thirdly, we evaluated transition between attractor states in terms of (in)stability and extraordinary events (cf. Figure 1).

### Analytical strategy

#### Summarizing daily life

The first step was to qualitatively analyze the anonymized daily records in accordance with the phased approach of thematic analysis [19]. This thematic analysis was conducted by the first author together with four Master’s students in Pedagogical Sciences, all under the supervision of a researcher with ample experience in qualitative methods. A thematic analysis is an inductive method whereby the coders collaboratively construct themes and patterns from the text in an iterative process that contains six phases: data familiarization, generating initial codes, searching for themes, reviewing themes, defining themes, and producing the research report. In each of these phases, the coders frequently came together to discuss and interpret the records. All five coders first familiarized themselves with the data by reading the whole daily records text file, which consisted of > 300,000 words. Together the coders then practiced the initial coding. The text file was then divided into five roughly equally large chunks of daily records text. Each coder then generated initial codes on his/her own text. The coding was done using MAXQDA 2022 [37]. During the initial code generating phase, coders came together thrice to compare each other’s initial coding wording and interpretation of the text. These iterative consensus-building sessions lead to the construction of a preliminary overview of candidate subthemes and themes (i.e., codes that were interpreted as reflecting the same higher-order construct). During this collaborative, inductive process, the wording and structure of these (sub)themes were refined into one thematic overview that contained a theme- and subtheme-structure that captured themes based on the whole dataset. This procedure fosters a shared understanding among all coders, resulting in a consensus over the overarching thematic structure (thematic map). From this jointly construed thematic map, every coder then coded the records once more from scratch. That finally resulted in a MAXQDA file with fragments of coded text on a specific day. These qualitative data were then quantified to a dataset containing only binary variables with a (sub)theme coding present (1) or not present (0) per day.

The researcher then met with the participant’s clinician, who has known her for over a decade, to discuss the thematic overview and underlying codes. The goal of this meeting with the clinician was to (1) ascertain the appropriateness of challenging behaviors as the most indicatory variables to summarize the system’s overall state, and (2) identify the most relevant (sub)themes for explaining the frequency of challenging behavioral incidents at any given period.

### Describing change trajectory

The subsequent steps were quantitative analyses – all performed in RStudio-2022.02.2–458 [38] which runs on R software version 4.2.0 [39]. To evaluate concurrent validity of self-ratings, we performed χ^2^ tests between self-ratings and informant-reported (daily records) accounts of days with self-injury and physical aggression. Kazdin [40] recommends evaluating single-case timelines by combining visual inspections of graphed timeseries with statistical analyses. We therefore visualized the two self-report timeseries (physical aggression and self-injury) using functionality from *ggplot2* [41].

Next, we pinpointed transitions in the physical aggression and self-injury timeseries on the 560-day timeline. This transition-point detection was done with the *ts_levels* function from package *casnet* [42], which uses recursive partitioning [43] to classify segments (or phases) on a timeseries with a relatively stable mean. We did this for the physical aggression and self-injury variables. Because these two variables are binary (0 = behavior did not occur on that day; 1 = behavior occurred on that day), mean levels effectively reflected the proportion of days with incidents within a phase. In the *ts_levels* function the minimum duration of one phase was set to seven days, comprising a whole weekly routine, and controlling for day-of-the-week effects. The absolute change criterion was set to 25%, meaning that each identified transition reflected at least a 25% increase or decrease compared to the mean of the preceding phase (cf [44, 45]). Based on suggestions by Kazdin [40], we searched for transitions by visually inspecting a graph of the raw binary timeseries and a plot of the levels identified using the *ts_levels* function [42].

After pinpointing transitions, we characterized the different attractor states in terms of what makes them (dis)similar from one another on the 560-day timeline. We calculated – per phase and across the whole 560-day timeline – the mean frequency of self-rated challenging behaviors (i.e., mean days with challenging behaviors) and the mean frequencies of (sub)themes that the participant’s clinician hypothesized to be explanatory. Furthermore, we examined – per phase and across the whole 560-day timeline – whether these clinically relevant (sub)themes were associated with challenging behaviors. That is, Fisher’s exact tests evaluated whether a reported challenging behavior occurred (beyond chance) on the same days as reports of staff-hypothesized risk- or protective factors. Additionally, we performed Fisher’s exact tests to evaluate the relation between staff-hypothesized risk- or protective factors from one day until the next (lag-1 association). Due to the number of repeated bivariate associations we evaluated significance at *p* < 0.01.

### Change-mechanisms

For the third and last step we analyzed temporal instability and pinpointed extraordinary events, to obtain insight into potential change-mechanisms (i.e., either instability-induced, event-induced or both). The (in)stability of daily self-ratings was analyzed with dynamic complexity [46] as implemented in R-package *casnet* [42]. Dynamic complexity is comprised of a multiplication between distribution measure D, which reflects the distribution uniformity of data-points within the range of the used scale, and fluctuation measure F, which indicates the strength and number of fluctuations within the timeseries. As such, it is more robust to non-stationarity and periodicity than alternative measures such as variance (cf [33, 46]). Because dynamic complexity cannot handle missing data, we first employed Kalman smoothing with the *na_kalman* function [47] to impute missing data-points using a structural model fitted by maximum likelihood. Dynamic complexity can only be computed for ordinal or continuous timeseries [46], hence dynamic complexity could not be computed for the binary variables aggressive and self-injury incidents. Instead, dynamic complexity was calculated on the most relevant six-point scale items: “urge for aggression” and “urge for self-injury”, each within a seven-day backwards overlapping window. This window shifts gradually along the timeseries without changing in width, such that dynamic complexity is first calculated for each item between day 1 and day 7, then between day 2 and day 8, and so on. With this 7-day window we again control for day-of-the-week effects. The windowed dynamic complexity was visualized on a timeline per item. A one-tailed *z* test (α = 0.05) was applied on each dynamic complexity timeline to determine at which time-windows there was significant instability (i.e., high dynamic complexity). We chose to perform a one-tailed significance test because we wanted to examine the occurrence of high dynamic complexity values (not low values), exceeding the threshold of the average dynamic complexity (cf [17, 18, 33]). We ultimately described, per identified transition, whether it was preceded or accompanied by significant instability and/or an extraordinary event. These extraordinary events were codes categorized into subthemes during the thematic analysis procedure. That is, after the coders had familiarized themselves with the data, generated and discussed initial codes they reached consensus about which events reflected everyday events and which events were extraordinary across the 560 day period. In the absence of instability, an extraordinary event occurring the week prior to a transition was considered an potential indicator of an event-induced mechanism (cf. Figure 1). Change was potentially instability induced when the dynamic complexity of aggression and/or self-injury was significantly high the week before or during change, without an extraordinary event the week prior. In the presence of both instability and an extraordinary event, we conclude change was potentially event- and instability-induced.

## Results

### Summarizing daily life

**Fig. 3:**
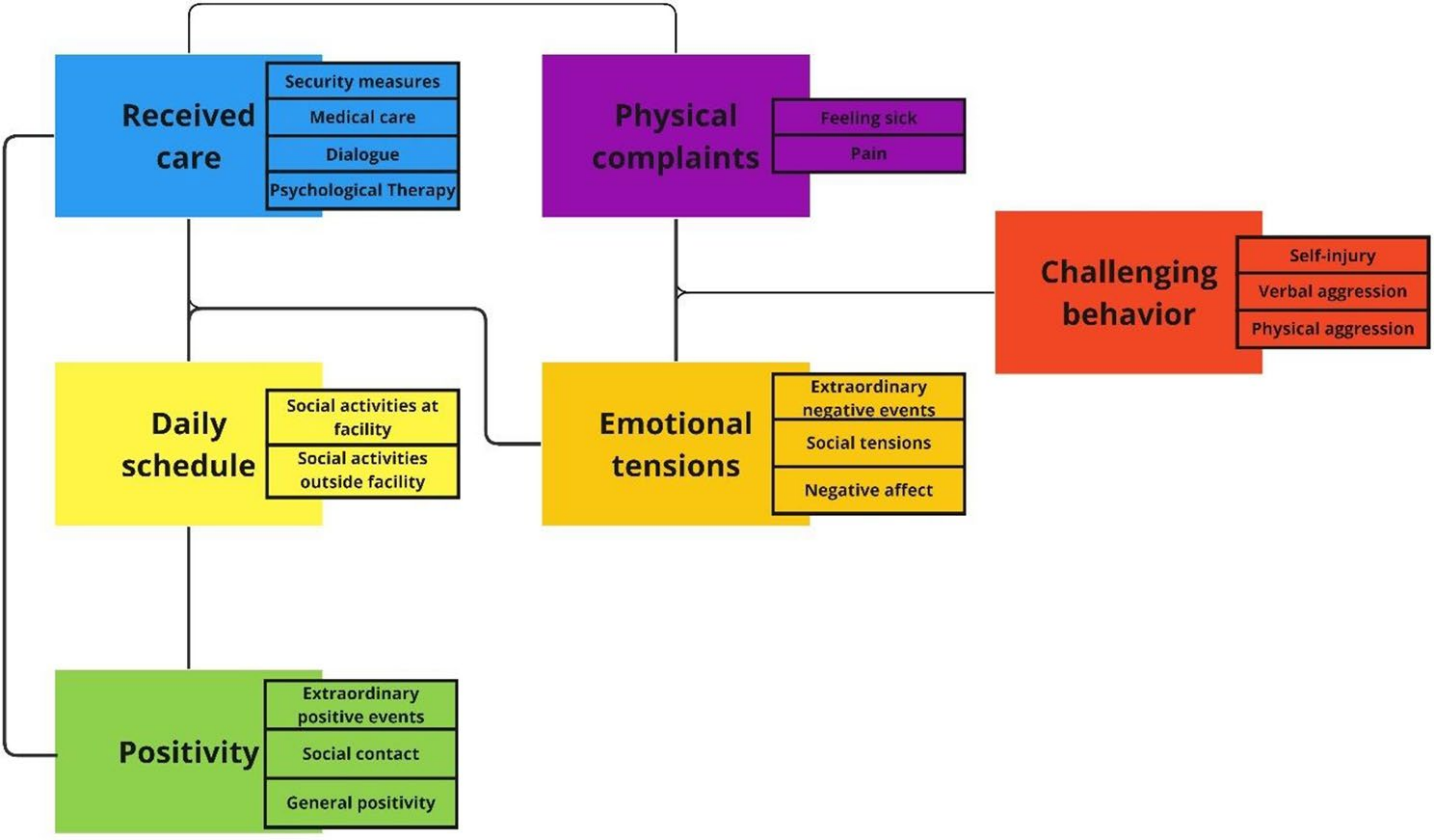
Thematic map, generated from the thematic analysis, showing (sub)themes and the links between them

To first obtain a comprehensive summary of her daily life we conducted a thematic analysis of the care taker’s records. The analysis resulted in a thematic map consisting of six themes and sixteen subthemes. The six themes were the received care, daily activities not related to care, positivity, physical complaints, emotional tensions, and challenging behavior. These themes reflect categorizations that are interrelated. We visualized (sub)themes and their interrelations in Fig. 3.

Anything positive reported in the daily records was coded under the theme *positivity*. This pertained to events that were extraordinary positive for her on the 560 days. Positive social contact was a subtheme that reflected more casual positive interactions with care professionals, family or friends. The subtheme general positivity included any mention of positive affect. This could be sense of humor, making a relaxed impression, having a good day, or positive dialogue with care professional. For example the mention “*client played boardgames after the barbecue and visibly enjoyed herself*” indicates that positivity occurred during descriptions of the issue of the day, which are subdivided under two themes: *received care* and *daily schedule.* The latter involved her daily schedule unrelated to medical or psychological treatment, which could be either at the facility (e.g., doing the household or taking a walk) or social activities away from the facility (e.g., board games at activity center). Received care related to any actions from care professionals, which could be either in the form of security measures (e.g., checking her room for potential objects used for self-injury or secluding the participant), dialogue with care professionals (e.g., talking about what is on her mind, complimenting the participant), medical care (e.g., treatment of wounds at care facility or hospitalization), or psychological therapy sessions (dialectical behavior therapy and psychomotor therapy).

*Challenging behavior* was a theme with three subthemes: verbal aggression, physical aggression, and self-injury. The latter two were also self-reported on a daily basis by the participant. Daily record accounts of challenging behavior related to emotional and/or physical discomfort, for example “*client cut herself with a broken piece of plate, she says she wanted to experience different pain than the pain in her stomach*”. The theme *physical complaints* related to either feeling sick (e.g., nauseated) or mentions of the participant communicating experiencing physical pain. Both could be a cause and consequence of challenging behavior. For example, self-injury caused wounds, which lead to inflammation, which naturally come with pain or sickness such as fever. Self-injury through re-opening existing wounds was the most frequently reported self-injurious form, which exacerbated physical complaints. That required her receiving (extra) care. Related to both challenging behaviors and physical complaints were *emotional tensions* – a broad theme that comprised of three subthemes. Records describing extraordinary negative events (e.g., losing her pet), social tensions (e.g., quarrels with staff or family) and general descriptions of negative affect (e.g., feeling irritated, fearful, frustrated, or insecure). Emotional tensions could be triggered during any daily activity and could be both cause and consequence of physical complaints. For example “*client is working on a painting. When we adjust schedule to playing a boardgame she becomes angry”*. Moreover, it could result in receiving extra care (e.g., support from staff when in distress) or was the consequence of dissatisfaction with received care (e.g., anger after imposed security measure). Challenging behavior always came with some form of emotional tension.

To better interpret the thematic map, the researcher then asked the participant’s clinician whether the participant knows better and worse times and what typically indicates to staff whether her overall well-being is high or low. Before having seen the results, she confirmed that the frequency of self-injurious and physically aggressive incidents is most telling about her overall well-being. This indicates challenging behaviors summarize her overall state. From the (sub)themes generated in the thematic analysis, the clinician then identified 11 staff-hypothesized risk- and protective factors for her challenging behaviors. These factors were either specific codes or broader (sub)themes: reliving past trauma, hallucinating, negative affect, receiving medical care, receiving compliments, the imposing of freedom restricting measures, experiences of physical pain and sickness, receiving psychological therapy, tensions with her family, and positive social interactions. These variables were used for subsequent analyses.

### Describing change trajectory

The participant completed the daily survey 494 times during the 560 days (88%). Physical aggressive incidents were self-reported on 65 days (13%), while self-injury was self-reported on 247 days (50%). Staff reported aggressive and self-injurious incidents on respectively 75 days (16%) and 164 days (33%). A χ^2^ test indicated agreement between self- and informant ratings. That is, counts of observed matches between self- and informant ratings of these challenging behaviors (i.e., both reporting daily presence or absence of behavior) was significantly higher than the expected count for self-injury, χ^2^ (1, *N* = 494) = 91.56, *p* < 0.001, and for aggression, χ^2^ (1, *N* = 494) = 12.76, *p* < 0.001. As both challenging behaviors can occur without being noticed by staff (e.g., when on leave), we analyze self-reported challenging behavioral dynamics.

**Fig. 4:**
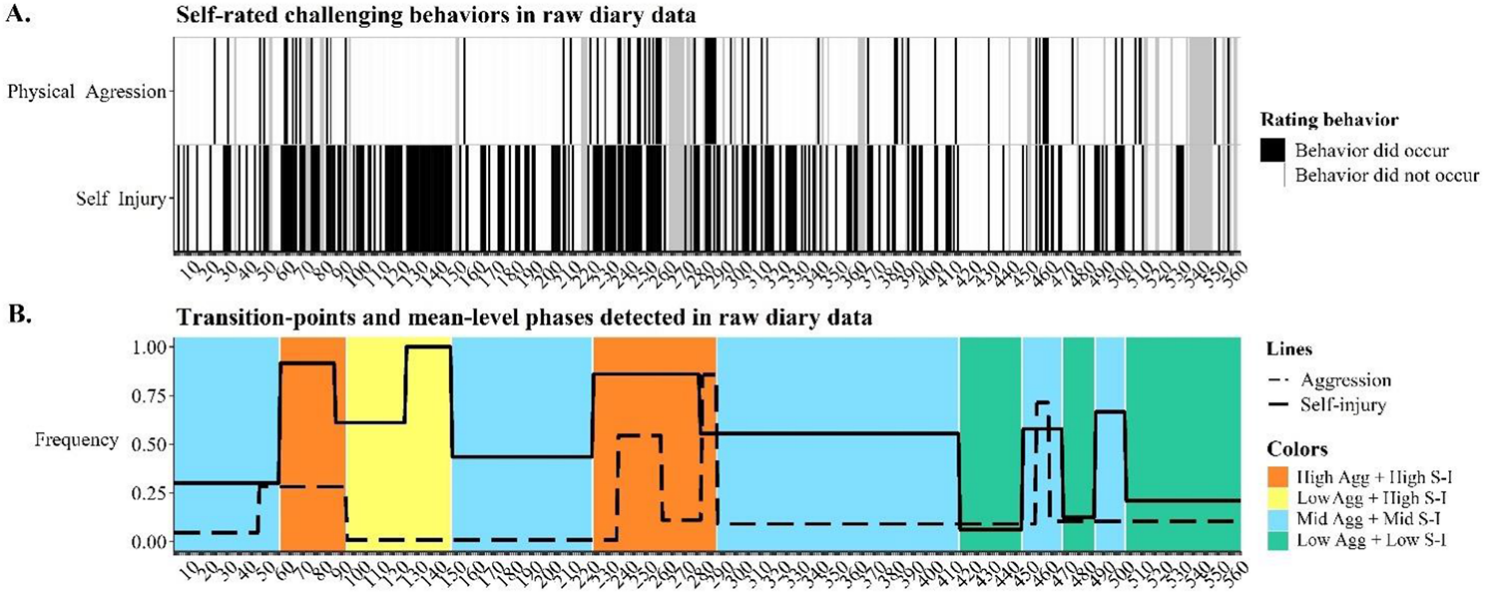
Binary timeseries of self-reported physical aggression and self-injurious behavior during 560 days and changes in its mean frequency. X-axes show number of days. Panel A shows raw challenging behavioral timelines. Gray cells are days that the participant did not complete her diary. In panel B, the lines reflect mean-level changes in raw diary timelines, detected by recursive partitioning algorithm. Colors reflect identified challenging behavioral attractor states (see text for details). The same color means a qualitatively similar attractor. Identified transition-points between attractors are thus the days (on x-axis) when the color changes

Figure 4A illustrates the raw binary timeseries of self-reported physical aggression and self-injury for 560 days. The recursive partitioning algorithm [42] first detected mean-frequency changes in raw diary timelines (4A) – the outcome of which is visualized with dashed and solid lines in Fig. 4B. After visual inspection of the binary timeseries (4A) and their mean-levels (lines in 4B), we found 10 transitions that mark that end of an old- and start of a new attractor (colors in 4B). When the mean-level changes detected by recursive partitioning (up or downward trend in lines 4B) of the two challenging behaviors occurred in the same direction within close proximity to one another (i.e., within 14 days), we marked it as transition that starts or ends a challenging behavioral phase. For example, on day 86 for self-injury a 30% drop was detected by recursive partitioning and on day 91 aggression dropped by 28%. Here we marked day 91 as the transition, as it marked the end of a phase with frequent challenging behavior. Similarly, when self-injury and aggression increased on respectively day 446 and 452, we marked 446 as the transition for a start with frequent challenging behaviors. One exception was made, based on a clear difference in absolute change: on day 46 the proportion of aggressive incidents increased with 25%, while 11 days later the proportion in self-injurious incidents increased by 60%. Hence, only day 57 was marked as a transition. Two detected mean-changes were not marked as transitions: the increase of self-injury on day 122 and the decrease in aggressive incidents on day 257. The latter (day 257) was not marked as an attractor change, because of the large number of missing values that followed this transition (see gray band in Fig. 4A). Day 122 was not marked after visual inspection of the self-injurious incidents timeseries (Fig. 4A) we noted that (1) the upward trend may have started sooner (possibly day 110) and (2) this upward trend did not seem significant as the frequency of self-injuries– relative to the entire timeline – was already high between day 57 and day 146.

Table 1 summarizes, for each phase, the mean frequency (i.e., percentage of days) that both challenging behaviors were self-reported in the diaries. Furthermore, we calculated the mean frequency per phase for each of the 11 staff-hypothesized risk- and protective factors (see Supplementary Material 1). To obtain insight into what makes phases (dis)similar from each other in terms of these risk- and protective factors, we compared the mean frequency of them within each phase to the 560-day mean of that factor. We considered a phase-mean salient if it was above or below 1 *SD* relative to that factor’s 560-day mean. For example, salient about phase 1 (day 1 to 56) was that familial tensions occurred on 18% of days, which was relatively often, given that it is > 1 *SD* relative to the 560-day mean of 5%. Although Table 1 shows that the 11 frequencies of staff-hypothesized risk- and protective factors differ between phases, we find no unequivocal bivariate if-then explanation (e.g., if a phase has familial tensions, then high aggression) for either of the challenging behavioral frequencies.

In addition to describing average frequencies across phases, we also analyzed bivariate associations at the within-day level (contemporaneous) and across days (lag-1). That is, whether challenging behaviors and reports of staff-hypothesized risk- and protective factors co-occurred on the same day and from day-to-day. Fisher’s exact test revealed that, across the entire 560-day timeline, freedom restricting measures were more often applied on days with aggression (OR = 5.27, 95%CI [2.82, 9.78]) or self-injury (OR = 2.72, 95%CI [1.56, 4.89]). Across the 560-day period, there were no bivariate contemporaneous associations between challenging behaviors and reliving trauma, hallucinating, receiving medical care, compliments or psychological therapy, having pain, sickness, experiencing negative affect or familial tensions, or positive interactions. On days *after* an implemented freedom restricting measure, our participant was more likely to engage in aggressive (OR = 4.80, 95%CI [2.58, 8.86]) and/or self-injurious behavior (OR = 1.97, 95%CI [1.67, 3.39]). On days after a psychological therapy session (DBT or psychomotor therapy) she was less likely to engage in self-injurious behavior (OR = 0.36, 95%CI [0.15, 0.79]). To explore these associations within phases (and possible differences between phases), we repeated the same Fisher’s tests per phase, on both the contemporaneously and lagged timescale (484 tests; 11 themes × 2 behaviors × 11 phases × 2 timescales). The only significant associations that hold within certain phases evolve around freedom restricting measures, indicating that these measures were more likely to occur on the same day as aggression in phase 5, before days with self-injury in phase 7 and before days with aggression in phase 11. All other contemporaneous and lag-1 associations between challenging behaviors and the 11 variables that the clinician hypothesized to be explanatory, were non-significant (evaluated at *p* < 0.01 due to multiple testing).

**Table 1:** Summary of what makes different phases salient in terms of self-reported challenging behaviors and frequencies of risk- and protective factors

Phase	Aggression	Self-injury	Salient frequencies of risk- and protective factors
Phase 1 Day 1–56	Average (8%)	Average (30%)	Many positive social interactions and many tensions in family
Phase 2 Day 57–91	High (30%)	High (87%)	Few positive social interactions, high negative affect, many hallucinations, few feeling sick
Phase 3 Day 92–146	Low (0%)	High (78%)	Nothing salient
Phase 4 Day 147–233	Average (6%)	Average (43%)	Low negative affect, few physical pain
Phase 5 Day 234–285	High (41%)	High (80%)	No therapy
Phase 6 Day 286–412	Average (11%)	Average (50%)	Few positive social interactions, few therapy, often reliving trauma
Phase 7 Day 413–445	Average (3%)	Low (6%)	Few positive social interactions, low negative affect, often in pain, few medical care
Phase 8 Day 446–466	Average (26%)	Average (58%)	Often reliving trauma, often receives compliments
Phase 9 Day 467–483	Average (6%)	Low (13%)	Often receives medical care, compliments, and psychological therapy
Phase 10 Day 484–499	Average (13%)	Average (67%)	Many positive social interactions, many tensions in family, many hallucinations, often sick and in pain
Phase 11 Day 500–560	Average (13%)	Low (21%)	Many positive social interactions, often sick

Note. Average frequency for each variable per phase were compared to the average for the entire 560 days. Salient interpretations (e.g., low, high, often, few) indicate that the mean of a phase is either > 1 *SD* or < 1 *SD* relative to 560-day mean of that variable

**Fig. 5:**
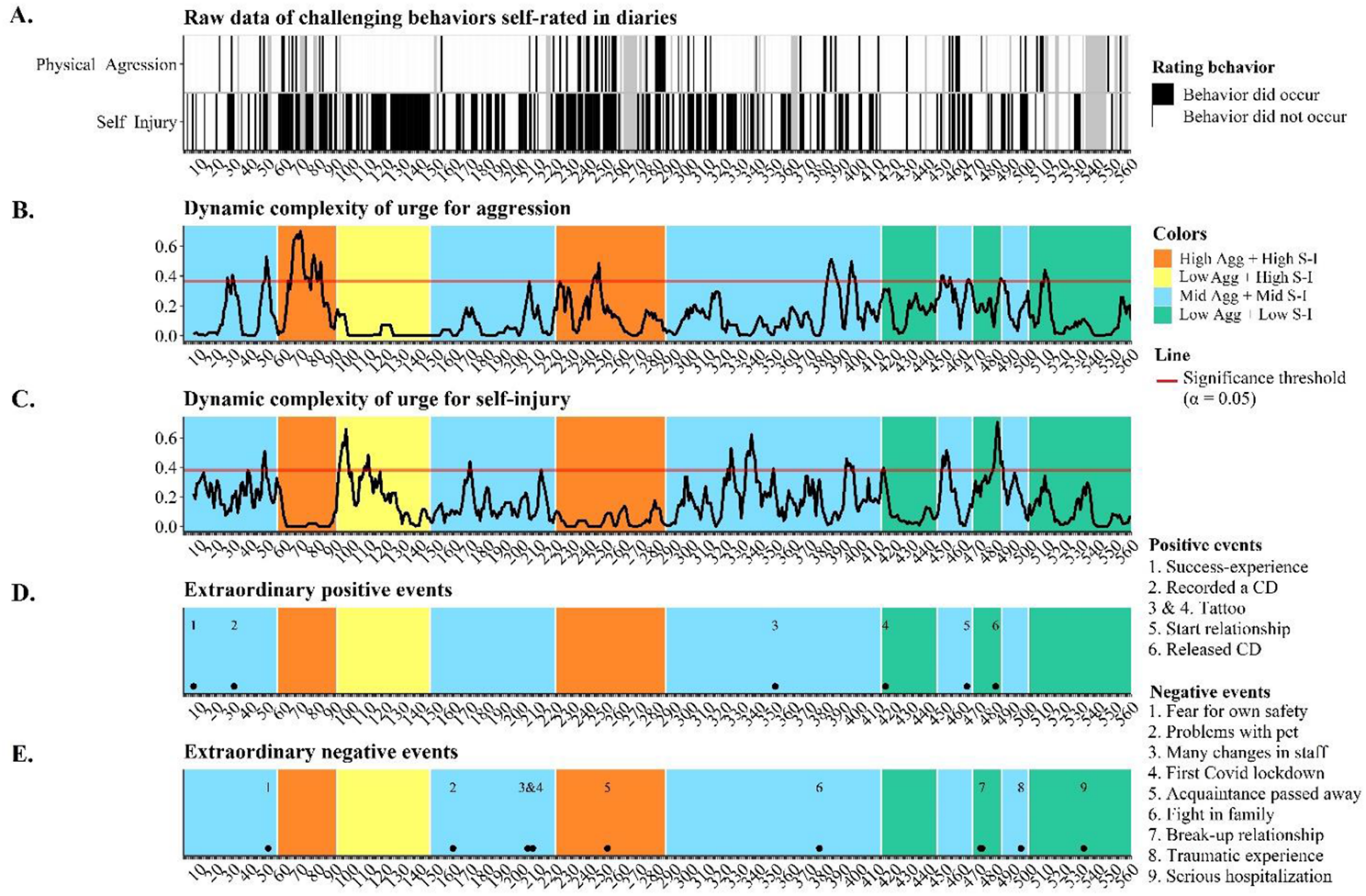
Combined graph of the participant’s self-reported challenging behavioral patterns, transition-points, dynamic complexity, and extraordinary events. Panel A shows the raw data of self-reported physical aggression and self-injury. Gray cells are missing data. Panel B and C reflect the dynamic complexity of both challenging behaviors. High values reflect unstable patterns, whereas low dynamic complexity reflects stability during the 7 prior days. The horizontal red lines mark the significance threshold for each variable; dynamic complexity values above the lines indicate statistical significance (α = 0.05). Orange, yellow, blue, and green background colors are attractor states. Panel D and E reflect pinpointed positive and negative extraordinary events that were identified as such in the daily records

**Table 2:** Summary of what happens the week prior to and during transitions in terms of instability and extraordinary events

Transition-point	Direction of change	Extraordinary event in the week prior to transition	Instability the week before or during change	Potential mechanism
Day 57	Undesirable	Fear for an escapee (negative)	Yes	Event- and instability-induced
Day 92	Desirable	None	Yes	Instability-induced
Day 147	Desirable	None	No	Unknown
Day 221	Undesirable	First Covid lockdown (negative)	No	Event-induced
Day 286	Desirable	None	No	Unknown
Day 413	Desirable	Finishes tattoo (positive)	Yes	Event- and instability-induced
Day 446	Undesirable	None	Yes	Instability-induced
Day 467	Desirable	Starts relationship (positive)	Yes	Event- and instability-induced
Day 484	Undesirable	Ends relationship (negative) then release of own CD (positive)	Yes	Event- and instability-induced
Day 500	Desirable	Traumatic experience (negative)	No	Event-induced

Note. Desirable change is towards an attractor with more frequent challenging behavior than before, and undesirable is the opposite. Extraordinary event is based on negative and positive event subthemes from thematic analysis. There is instability when the dynamic complexity value of one or both challenging behaviors were significantly high, based on a *z*-test with α = 0.05. The last column shows whether our findings indicate what was a likely change-inducing mechanism. We conclude that change was potentially instability induced when the dynamic complexity of aggression and/or self-injury was significantly high the week before or during change, without an extraordinary event the week prior. Event-induced change is when there was an extraordinary event the week prior but no significant instability the week before or during change. In the presence of both instability and an extraordinary event, we conclude change was potentially event- and instability-induced

### Change-mechanisms

Figure 5 shows the occurrence of challenging behaviors (panel A), the (in)stability of self-reported patterns in urges for challenging behaviors (panel B and C), and extraordinary events (panel D and E) on the 560-day timeline^1^. Each point on the graphs in panel B and C reflects how unstable (i.e., irregular and erratic) the fluctuations of self-rated urges for challenging behaviors were in the previous 7 days. Low values indicate stable patterns, whereas high dynamic complexity values are indicative of temporal instability. Everyday events are extremely plentiful, making them impractical to pinpoint on a timeline. Extraordinary events, however, were derived from thematic analysis results. We considered two subthemes: positive events and negative events (see themes positivity and emotional tensions in Fig. 3), as they reflected impactful events that were extraordinary across the 560-day timeline.

Table 2 summarizes what happens one week before each of the 10 transition-points. There were four transitions towards an attractor with more frequent challenging behavior than before. These undesirable transitions were all either instability-induced, environment-induced or a combination of both (Table 2). Day 221, for example, was likely an event-induced change, given that there was no instability, but the first Covid-19 lockdown likely led to this undesirable change. Social contact with friends and family – as well as support from staff – were drastically reduced while in lockdown, disrupting her everyday routine increasing her need for aggression and self-injury as an outlet. There were also six desirable changes. One such example was that the week before she finished her tattoo (extraordinary event on day 413) was instable, possibly due to prospect of this exhilarating moment, marked the start of a new phase with few challenging behaviors. However, the relation between transitions, instability and extraordinary events was not entirely clear-cut, as two desirable transition-points (day 147 and 286) occurred during stable periods and without any notable events. Figure 5 further shows that extraordinary events occurred during stability, but without a transition (e.g., starting her tattoo on day 350). Even an extraordinary event in combination with instability was no guarantee for a transition (e.g., on day 367 a fight in the family occurred during a highly unstable week without a transition). In summary, although instability seemed to increase the chance of transitions – especially in combination with an extraordinary event – our findings do not imply that instability and extraordinary events are incontrovertible warning signals that always explain meaningful change on the participant’s 560-day timeline.

## Discussion and conclusions

The current study provides a unique exploration of day-by-day aggressive and self-injurious patterns in one woman with a MID and BPD. Applying a three-step-approach inspired by complex systems theory, we aimed for an in-depth understanding of her challenging behaviors over the course of 560 days. Summarizing her daily life was the first step, revealing that a large set of internal and environmental factors relevant to her daily life. The clinician narrowed this large set down to 11 staff hypothesized risk- and protective factors: freedom restrictive measures, reliving trauma, hallucinating, experiencing pain, sickness, negative affect, familial tensions, positive interactions, receiving medical care, compliments or psychological therapy. Overall, freedom restricting measures were more likely to occur on the same day as challenging behaviors, which is not surprising. It is striking, however, that self-injury and/or aggression were more likely to occur the day *after* a coercive measure by staff, indicating that although these measures may be effective to suppress certain behaviors in the moment, they have detrimental effects on the longer run [4, 5]. Furthermore, we found that on the day after a psychological therapy session (DBT or psychomotor therapy) she was less likely to self-injure. These results imply that downscaling of freedom restricting measures and upscaling of psychological therapy (where possible) is warranted. All other bivariate associations between hypothesized risk- and protective factors with both challenging behaviors – explored phase-by-phase and day-by-day – were non-significant, indicating that challenging behaviors are not governed by mono-causal if-then explanations (e.g., if phase has many familial tensions, then high aggression or if day with hallucination, then self-injury). The multitude of bivariate null-results speaks to the complex nature of these behaviors at the case-level [2, 6–8].

In the second step, we described the trajectory of challenging behaviors over time. We identified 11 distinct, relatively stable phases within the 560-days timeline. These 11 phases could be narrowed down to four qualitatively different attractor states: high levels of self-injury and aggression (2 phases), average levels of self-injury and aggression (5 phases), low levels of self-injury and aggression (3 phases), or high levels self-injury with low levels of aggression (1 phase). The mean frequency of the 11 staff-hypothesized risk- and protective factors varied by phase: no two phases were similar (Table 1).

In the third step we focused on (the week before) transitions between attractors, exploring potential change-inducing mechanisms (Fig. 1). Our findings suggest that the mechanism of two transitions remained unknown, two were event-induced, two were instability-induced and four could be environment- and/or instability-induced (Table 2). Six transitions were thus potentially instability-induced, which is in line with empirical evidence for instability as an early warning signal for upcoming transitions [17, 33, 34]. Nevertheless, extraordinary events and/or instability did not unequivocally imply a transition, as both instability and extraordinary events occurred without transitions afterwards (Fig. 5). The two unknown mechanisms were both for desirable transitions, which could mean that relatively minor events in daily life apparently were enough to elicit positive change. One possible explanation would be that her desirable attractor is stronger than the undesirable one. That is, we could perceive her undesirable basin (Fig. 1) to be shallower, making this state easier malleable relatively minor everyday events. Future research could explore this further with recently developed analytical methods that quantify the stability of an attractor state [48].

There were three notable limitations to this study. First, results from a case-study are obviously not generalizable. Repeating (and finetuning) our three-step-approach on different cases, will reveal the extent to which of our findings are person-specific or generalizable across cases. This will ultimately increase our understanding of challenging behaviors and consequently enable optimized care. Second, our thematic analysis was based on care professionals’ daily records. Registering relevant events in the electronic health records is a routine practice in the residential care setting – done with the intention to document the client’s case file and keep colleagues up to date. Hence, care professionals received no instructions as to how extensive or comprehensive their reports should be. This meant that when a specific code was not identified from the records on a specific day, it may either have not been observed by care professional(s) or simply not been registered. Seemingly trivial happenings, such as giving complements will likely have occurred more often than that the coders coded in the records. Third, despite a-priori anonymization of the records, it was evident that the records included reports of many different (approximately > 30 different) care professionals. The richness of the described daily events likely partially depended on who reported and how much time that person had. Fourth, our three-step procedure was subject to many researcher’s degrees of freedom. The 11 staff-hypothesized (sub)themes that the participant’s clinician selected out of the thematic map, for example, remained a personal choice. Furthermore, the criterion we used to evaluate a threshold for instability (one tailed z-test at *p* < 0.05) is based on convention (cf [16, 18, 33]), but ultimately still a choice. On the other hand, there are no established guidelines available for a complex systems guided case study.

This study also had strengths. First, by shedding light on events in the environmental that may ‘push’ the system into another state, our study adds to the (complex systems) psychological literature that has so far predominantly focused on instability preceding transitions [24, 33, 34, 45]. Qualitative analyses of case records allowed us to distinguish everyday- from extraordinary events. Because this distinction was informant-based and not self-reported, it is possible that meaningful events were missed (here or in any step of our analysis). Future qualitative or mixed-methods research should further explore the nature of events that the individual perceives to ‘kickstart’ transitions. A second strength is that our research gives a helicopter view of day-by-day processes across several months. The majority of EMA research in BPD studies within-day fluctuations. For our participant behavior did not only fluctuate within-days, also across time-periods of multiple weeks or months. This may inspire EMA research in BPD to consider further exploring fluctuations on slower timescales. Nevertheless, within-day processes remain relevant. Complex systems, after all, are characterized by interacting processes across many timescales [12, 49]. In our case, unobserved instability at shorter timescales (e.g., hour-to-hour) could have induced our (un)observed transitions. After all, within-day affective instability is a well-documented correlate of challenging behaviors in BPD [23, 24]. The case records did provide within-day detail, but because we eventually quantified these into dichotomous codes per day (present vs. absent), the richness of within-day information was lost. Future research should zoom further into what happens within the day of (or days before) a transition. Statistical process control charts [50] could then be used to detect whether significant rises in tensions predict challenging during the day.

The participant selection in this study was solely based on convenience sampling, that is, she was the only one in DBT who adhered to the diaries this consistently for this long. The uniqueness of the already collected diary data, both in terms of the chronicity of her challenging behavior [28, 29] and her devoted compliance to the diaries, was the reason she and her legal guardians were asked for this study. Whether or not these study procedures can be replicated in different cases depends on how well the implemented diary procedure elicits an intrinsic motivation to stay compliant. There were certain participant- and study characteristics that contributed to her uniquely long-term compliance, which are lessons for scientists or practitioners who wish to collect similar data. First, the diaries were an integral part of her DBT program – for which she was already highly motivated. Second, the diaries items were constructed in collaboration with the participant, and thus tailored to her experience world. A personalized approach to EMA in practice, by integrating it in therapy and individualizing item-selection, is an opportunity for increasing participant involvement and compliance [22, 51]. Third, for compliance it may have been helpful that the participant has lived in residential care since childhood. This institutionalization – at least with our participant – contributed to the responsibility she felt to follow through on prescribed activities in her care plan. Completing the diaries became part of her daily routine structure. It is likely that this played a part in her continued compliance to the diaries, even when the Covid pandemic made DBT impossible. Nevertheless, further research into factors that enhance or hamper EMA compliance is necessary.

Importantly, personalized daily diary monitoring – and therefore this study’s three-step analytical procedure – is already certainly feasible for other individuals [22]. Replicating this design is therefore encouraged. Complex systems theoretical principles have already guided mainly quantitative timeseries analytical inquiries in different clinical case studies with less measurements (e.g., 91 [17] or 138 [18]) and more measurements (e.g., 1.476 [15]). Based on these studies [15, 17, 18] we would we expect that altering between different phases over time is a finding that is likely to replicate. However, other clients without such chronic challenging behavior and without such an institutionalized background would likely show very different patterns. That is, dynamic patterns with qualitatively different – and potentially less strong – attractor states. At this point, it remains speculation how this case study’s findings relate to other clients. The surge of EMA applications in clinical settings during the past years suggests that large *n* = 1 datasets may become more commonly available. Replicating our three-step method would allow for between-person comparisons, shedding light on how (a)typical the nature of our participant’s attractor states and number of change-points was, compared to others (e.g., people with BPD and/or in residential MID care).

The study altogether illustrates the added value of in-depth case-study research [9] and the utility of complex systems principles to guide such an inquiry. Our three-step approach adheres to recent calls for holistic and dynamic accounts of challenging behaviors in BPD [52]. Over time, few (if any) if-then relationships could be said to possibly explain the participant’s challenging behavior, substantiating it as a complex phenomenon that is difficult to grasp. Our results thus make explicit why care professionals describe to these behaviors as “complex” [6]. Nevertheless, in-depth idiographic science can help disentangle this complexity, generating new insights relevant for practice. Zooming out revealed different phases of challenging behaviors. For staff it is good to recognize available attractors and adjust care accordingly. With our participant it illustrated that she – just as anyone – has both ups and downs. Her desirable attractors actually emerged more often than desirable ones (three periods of low aggression and self-injury vs. two periods with high aggression and self-injury). Moreover, her desirable patterns were less easily malleable than undesirable ones. For the participant, this means that when things are down, keeping in mind better times are ahead is as hopeful as it is realistic. Repeating this idiographic design on other persons with chronic challenging behavioral patterns may therefore nuance the bad reputation they may have at the care facility.

### Electronic supplementary material

Below is the link to the electronic supplementary material.

Supplementary Material 1

Supplementary Material 2

## Data Availability

R scripts are publicly available from 10.17605/OSF.IO/XRMHU. Preprocessed data are available upon reasonable request via 10.17026/SS/VOXYE9. Requests can be made for research purposes only.

## Footnotes

1 In Fig. 5 we present the dynamic complexity of the most relevant two variables for these challenging behaviors. For completeness sake, we present raw data of all seven self-rated variables and their (average) dynamic complexity in Supplementary Material 2. Visualizations of the 11 staff-hypothesized risk- and protective factors, in combination with challenging behaviors and instability are accessible through https://hulsmans.shinyapps.io/themes/.
