# Supplementary material for: A complex systems perspective on chronic aggression and self-injury: case study of a woman with mild intellectual disability and borderline personality disorder
Source: BMC Psychiatry. 2024 May 21;24:378. doi: 10.1186/s12888-024-05836-7 (PMC11110386; doi:10.1186/s12888-024-05836-7)
Supplement: Supplementary file 1 — Supplementary Material 1 [file 12888_2024_5836_MOESM1_ESM.docx]

**Appendix A.**

*Mean frequencies of challenging behaviors and staff-hypothesized risk- and protective factors per phase.*

|  | Phase 1  Day 1-56 | Phase 2  Day 57-91 | Phase 3  Day 92-146 | Phase 4  Day 147-233 | Phase 5  Day 234-285 | Phase 6  Day 286-412 | Phase 7  Day 413-445 | Phase 8  Day 446-466 | Phase 9  Day 467-483 | Phase 10  Day 484-499 | Phase 11  Day 500-560 | Whole timeframe  Day 1-560  *M (SD)* |
| --- | --- | --- | --- | --- | --- | --- | --- | --- | --- | --- | --- | --- |
| **Theme Emotional tensions** | | | | | | | | | | | | |
| Relive Trauma | 0.04 | 0.03 | 0 | 0 | 0.05 | **0.21** | 0.06 | **0.33** | 0 | 0.19 | 0 | 0.08 (0.11) |
| Hallucinations | 0.02 | **0.17** | 0.09 | 0 | 0.05 | 0 | 0 | 0 | 0 | **0.25** | 0 | 0.05 (0.08) |
| Negative affect | 0.73 | **0.86** | 0.65 | **0.53** | 0.57 | 0.58 | **0.45** | 0.71 | 0.76 | 0.69 | 0.66 | 0.65 (0.12) |
| Tension in family | **0.18** | 0.03 | 0.02 | 0 | 0 | 0.06 | 0 | 0 | 0.06 | **0.13** | 0.08 | 0.05 (0.06) |
| **Theme Physical complaints** | | | | | | | | | | | | |
| Pain | 0.27 | 0.29 | 0.31 | **0.20** | 0.22 | 0.54 | **0.61** | 0.24 | 0.41 | **0.69** | 0.44 | 0.38 (0.17) |
| Sick | 0.20 | **0.14** | 0.33 | 0.28 | 0.15 | 0.31 | 0.27 | 0.33 | 0.29 | **0.44** | **0.46** | 0.29 (0.10) |
| **Theme Received care** | | | | | | | | | | | | |
| Medical care | 0.82 | 0.89 | 0.8 | 0.77 | 0.85 | 0.77 | **0.73** | 0.90 | **0.94** | 0.88 | 0.74 | 0.83 (0.07) |
| Freedom restricting measure | 0.07 | **0.60** | 0.13 | 0.07 | 0.18 | 0.18 | 0.09 | 0.19 | 0 | 0.13 | 0.05 | 0.15 (0.16) |
| Psychological therapy | 0.14 | 0.14 | 0.07 | 0.04 | **0** | **0.04** | 0.12 | 0.14 | **0.24** | 0.13 | 0.05 | 0.10 (0.07) |
| Received compliment | 0.14 | 0.23 | 0.15 | 0.14 | 0.12 | 0.12 | 0.15 | **0.33** | **0.35** | 0.19 | 0.15 | 0.19 (0.08) |
| **Theme Positivity** | | | | | | | | | | | | |
| Positive social contact | **0.27** | **0.06** | 0.24 | 0.12 | 0.14 | **0.09** | **0.09** | 0.19 | 0.18 | **0.31** | **0.31** | 0.18 (0.09) |
| **Daily self-ratings** | | | | | | | | | | | | |
| Self-injury | 0.30 | **0.87** | **0.78** | 0.43 | **0.80** | 0.50 | **0.06** | 0.58 | **0.13** | 0.67 | **0.21** | 0.48 (0.28) |
| Aggression | 0.08 | **0.30** | **0** | 0.06 | **0.41** | 0.11 | 0.03 | 0.26 | 0.06 | 0.13 | 0.13 | 0.14 (0.13) |

*Note.* Bolded frequencies are at least 1 SD above or below the mean frequencies of the whole 560-day period of that variable.
