# Supplementary material for: A complex systems perspective on chronic aggression and self-injury: case study of a woman with mild intellectual disability and borderline personality disorder
Source: BMC Psychiatry. 2024 May 21;24:378. doi: 10.1186/s12888-024-05836-7 (PMC11110386; doi:10.1186/s12888-024-05836-7)
Supplement: Supplementary file 2 — Supplementary Material 2 [file 12888_2024_5836_MOESM2_ESM.docx]

**Appendix B.**

*Combined graph of transitions, the participant’s self-reported challenging behavioral patterns and self-rated emotions, average dynamic complexity of all emotions, and extraordinary events.*


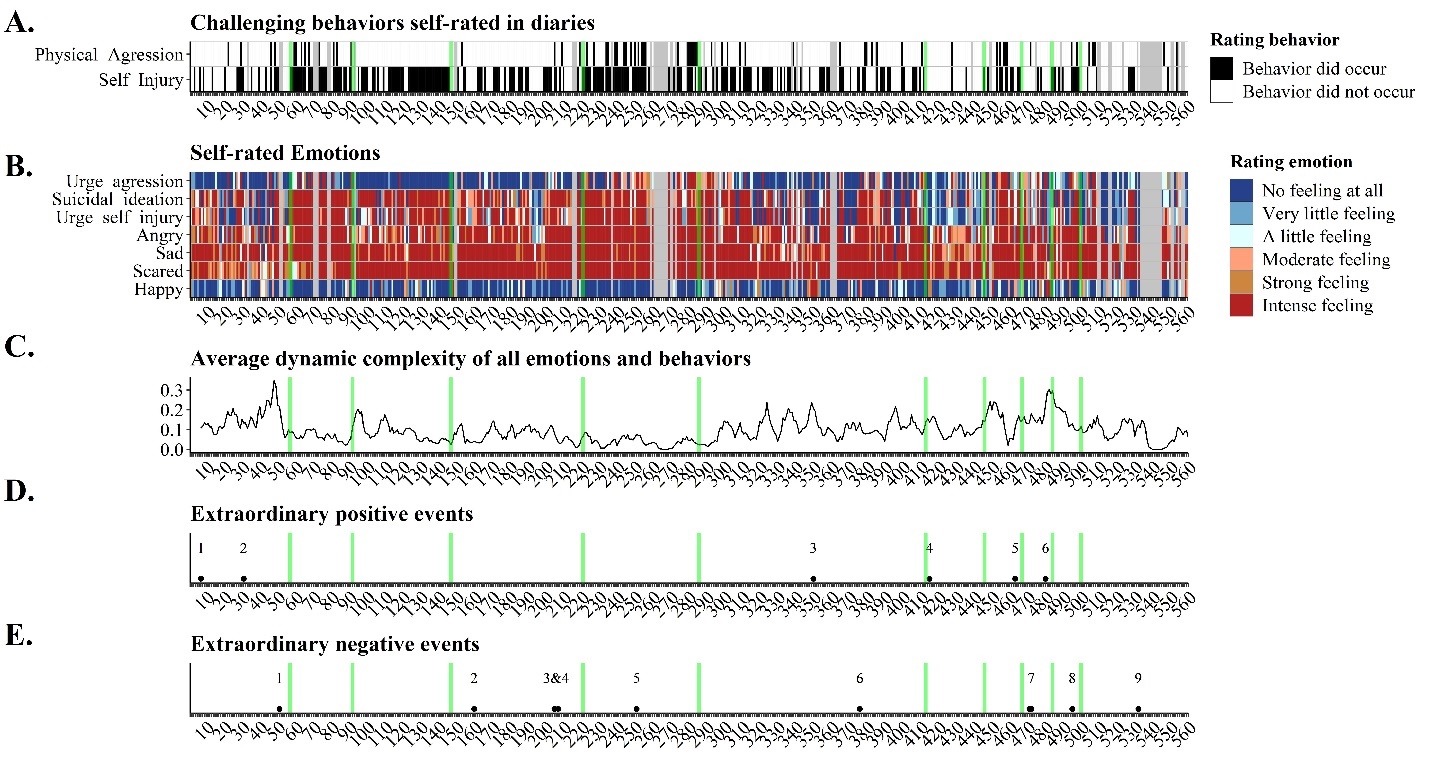
*Note.* Green vertical lines reflect identified transitions. Panel A shows the raw data of self-reported physical aggression and self-injury. Gray cells are missing data. Panel B is a raw data resonance diagram which reflects, on a colour-coded scale, the intensity of seven emotions. Panel C reflects the average dynamic complexity the emotions visualized in panel B. High values reflect unstable patterns, whereas low dynamic complexity reflects stability during the 7 days prior. Panel D and E reflect pinpointed extraordinary positive and negative events identified in the daily records.
